# A Comparison of the Results of Platelet-Rich Plasma Injection Versus the Results of Corticosteroid Injections in De-Quervain Tenosynovitis

**DOI:** 10.7759/cureus.61471

**Published:** 2024-05-31

**Authors:** Ahmed T Ashour, Ahmed Ashour, Abdelhamid H Khalaf, Ahmed A Marie, Emad A Mohamed

**Affiliations:** 1 Trauma and Orthopedics, Elhadara University Hospital, Alexandria, EGY; 2 Trauma and Orthopedics, Queen Elizabeth Hospital Birmingham, Birmingham, GBR; 3 Orthopedic Surgery and Traumatology, Faculty of Medicine, Alexandria University, Alexandria, EGY

**Keywords:** platelet-rich plasma, visual analog scale, de-quervain tenosynovitis, corticosteroids, injection

## Abstract

Background: De Quervain tenosynovitis (DQT) is a condition that affects the first extensor compartment of the wrist, resulting in stenosing tenosynovitis. This work aimed to evaluate the effects of platelet-rich plasma (PRP) injection in the treatment of DQT in comparison with corticosteroid (CS) injections.

Methods: This study was carried out on 40 DQT patients aged above 18 years old of both sexes, based on a combination of clinical symptoms and signs including persistent tenderness on the radial styloid, swelling on the radial styloid, positive provocative tests such as the Finkelstein test, and patients with failed medical treatment. Patients were divided into two equal groups: group I and group II. Group I was injected with PRP, and group II was injected with CS. Follow-ups were conducted at two weeks and six months.

Results: There were statistically significant differences among both groups regarding the visual analog scale (VAS), and Disabilities of Arm, Shoulder, and Hand (QuickDASH-9) score. However, complications were statistically insignificant between both groups. After injection, CS was better than PRP after two weeks, but PRP was superior to CS after six months concerning QuickDASH-9 and VAS. These differences were statistically significant.

Conclusions: CS is more effective than PRP in the short term (two weeks) and PRP is more effective in the intermediate term (six months). Both modalities are safe; however, PRP is relatively safer than CS.

## Introduction

De Quervain tenosynovitis (DQT) was first discovered by a Swiss surgeon Fritz de Quervain in 1895. It is a condition that affects the first extensor compartment of the wrist, resulting in stenosing tenosynovitis. The condition causes thickening of the sheaths that encompass the abductor pollicis longus (APL) and extensor pollicis brevis (EPB) tendons as they traverse through their fibro-osseous tunnel, which is located along the radial styloid [1]. De Quervain tendinopathy usually affects females, more than males, aged 30-50 years. Risk factors include overuse, such as knitting, sewing, dishwashing, and phone texting. It can also occur in post-traumatic or postpartum conditions [2]. Patients usually present with radial-sided wrist pain aggravated by thumb and wrist movement. Patients face difficulty in performing daily tasks such as opening a jar lid and lifting objects. Pain over the radial styloid and fusiform swelling are observed as well [3].

Diagnosis can be made clinically by the presence of radial-sided wrist pain along with swelling. Special tests such as the Finkelstein test, Eichhoff's test, and wrist hyperflexion and abduction (WHAT) test aid in the diagnosis. X-rays are of no diagnostic value but may show generalized signs and can aid in ruling out other causes of pain such as fracture and arthritis. Ultrasound and MRI scans are the diagnostic radiological modalities for these conditions [4,5]. The treatment regimens consist of non-operative methods and operative ones. The non-operative methods include immobilization and local injection [6]. The administration of CS injections for musculoskeletal ailments gained widespread acceptance during the 1950s. While the efficacy of this therapy is commonly due to the anti-inflammatory properties of corticosteroids (CS), the precise mechanism of action remains uncertain as histopathological analysis fails to reveal any evidence of inflammation [7].

In contemporary medical practice, platelet-rich plasma (PRP) injections are emerging as a viable option for the treatment of tendinopathies that have proven to be resistant to conservative management strategies [8]. Conservative management through non-steroidal anti-inflammatory drugs (NSAIDS), splinting, and injection are all methods proven to be effective as the first line of management of this condition. In case of failure of conservative management, surgery is usually performed as a last resort in an outpatient setting [9].

This work aimed to evaluate the effects of PRP in the treatment of De Quervain’s disease (DQD) in comparison with CS injection.

## Materials and methods

An informed written consent was obtained from the patients and their relatives. The study was conducted after approval from the Ethical Committee of Alexandria University Hospital. This prospective study was carried out on 40 DQT patients aged above 18 years old of both sexes, based on a combination of clinical symptoms and signs including persistent tenderness on the radial styloid, swelling on the radial styloid, positive provocative tests such as the Finkelstein test, and patients with failed medical treatment.

Exclusion criteria were history of rheumatic disease, history of trauma or fracture in the hand or the wrist joint, history of endocrinal diseases such as diabetes mellitus and thyroid disorder, pregnancy, previous CS injection for the treatment of DQD, and any shoulder or elbow problems that might lead to misdiagnosis. Patients were divided into two equal groups: group I and group II. Group I was injected with PRP, and group II was injected with CSs.

All patients were subjected to history taking (name, age, sex, affected side, occupation, and residence), complaints (radial-sided wrist pain [1st extensor compartment], exacerbation of symptoms on grasping and raising objects with the wrist in a neutral position, swelling at the radial side of the wrist, limitation of motion), present history (mode of onset, duration of symptoms, relation to trauma or special habits, and history of any systemic diseases as diabetes mellitus and rheumatoid arthritis), past history (previous trauma or arthritis), family history (rheumatoid arthritis, diabetes mellitus, and blood diseases], drug history (treatments received especially local CS injection), and general examination (local examination: side, movements, tenderness, swelling, and special tests).

Preparation of PRP

**Figure 1:**
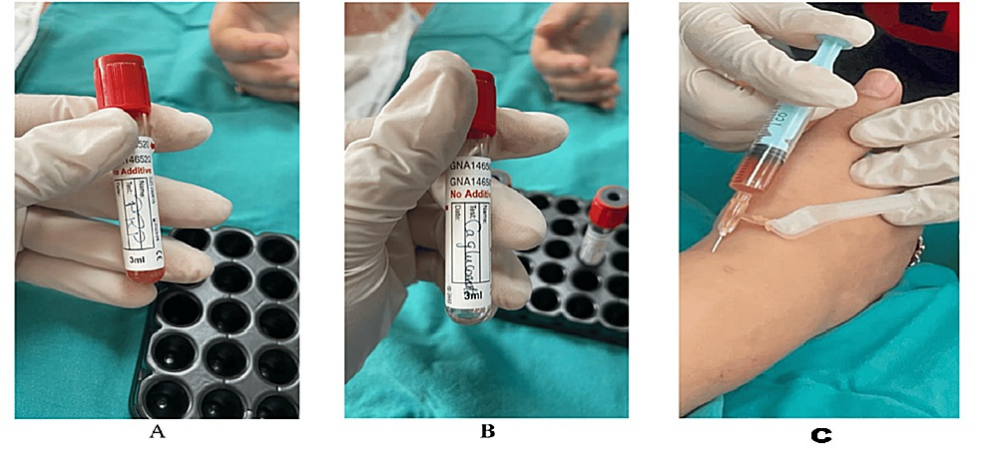
Preparation of PRP (A) Platelet-rich plasma after being prepared by double centrifugation of the patient's blood. (B) Calcium gluconate is used as an activator. (C) PRP injection technique: PRP is injected in the first compartment using the aseptic technique. PRP: Platelet-rich plasma.

About 20 ml of whole blood was obtained from each patient using a venepuncture system on acid-citrate dextrose (ACD) tubes. All steps were carried out in biological biosafety cabinet class II (Nuaure USA; NU-534-400E/2020). The blood was centrifuged using a "soft" spin at 200-250 g (1100-1200 rpm) for five minutes by DLAB swinging centrifuge (DLAB Scientific, USA; DM 0636). The supernatant plasma containing platelets was transferred into another sterile tube (without anticoagulant). The tube was centrifuged at a higher speed for a hard spin at 300 g (4000 rpm) for 15 minutes to obtain a platelet concentrate using the DLAB swinging centrifuge (DLAB Scientific, USA; DM 0636). At the bottom of the tube, the platelet pellets were formed. The supernatant platelet-poor plasma was removed, and the platelet pellets were suspended in a minimum quantity of plasma (1.5 mL) by a vortex. Activator consisting of 0.5 cm calcium gluconate was prepared (Figure 1).

Preparation of CS

The CS consisted of a single injection of 1 mL of CS combined with 0.5-1 mL of a local anesthetic. Water-soluble preparations were used.

Injection procedure

**Figure 2:**
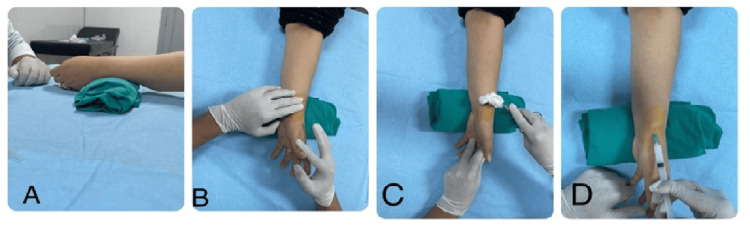
CS injection technique (A) The hand is placed on a pillow with slight ulnar deviation. (B) Tendons of the fist compartment are palpated. (C) The area is prepped sterile. (D) Then, the injection is administered at a 45-degree angle proximal to the radial styloid at the first compartment. CS: Corticosteroid.

The procedure began with step 1 in which the wrist was positioned in a neutral position with slight ulnar deviation, and the radial styloid was facing upward. Then in step 2, the injection site was prepped sterile. This was followed by step 3 in which the course of the APL and EPB tendons along the radial styloid was palpated, and extending and abducting the thumb made the tendon more prominent. In step 4, a 10-gauge needle was introduced at 45 degrees from distal to proximal into the tendon sheath at the level of the styloid, parallel to the tendons. Resistance indicated that the needle was likely in the tendon. Then in step 5, the needle was carefully backed out while maintaining pressure on the plunger of the syringe. Finally, in step 6, visual and palpable inflation of the compartment was noticed, denoting the smooth flow of the medication (Figure 2).

Assessment of patients’ satisfaction using QuickDASH-9

The 30-item DASH questionnaire was commonly used to assess upper limb functional restrictions. As a trial to augment practicality and enhance the speed and efficacy of the process, the 11-item QuickDASH was developed and offered a faster, more practical way to assess patients’ satisfaction. However, the validity of the QuickDASH-11 was questioned as the results for factor structure are conflicting. The QuickDASH-11 was considered invalid by several authors as its bi-dimensional structure made a single summated score inappropriate. The QuickDASH-9 consists of nine questions. Each item is scored from 0-4, instead of the 1-5 score used in the QuickDASH-11. A minimum of eight questions must be answered for the score to be valid. To calculate the QuickDASH-9, the sum of responses is multiplied by 1.1, and the result is multiplied by 5/2. A minimum score of 0 to a maximum score of 100 is obtained [10,11]. Based on the patient’s observation in this study, patients were classified as mild disability (0-30), moderate disability (31-65), and severe disability (66-100) based on minimal upper limb pain, having pain but working, and being unable to work due to upper limb pain.

Assessment of the extent of pain using VAS

Visual analog scale (VAS) is a pain scale scoring system. Patients subjectively quantify their symptoms via a single mark placed on a score sheet from 0-10. With 0 being no pain at all on the left end of the scale and 10 being the worst pain on the right end of the scale [12]. Measures are reported in centimeters and represent the severity of their pain. These numbers can be utilized to track the progress of their symptoms and compare the degrees of pain between patients with similar pathologies [13]. Complications such as fat atrophy, post-injection flare, hypopigmentation, neuritis, and tendon rupture were monitored.

Statistical analysis

Statistical analysis was done by SPSS v28 (IBM Corp., Armonk, NY). Shapiro-Wilks test and histograms were used to evaluate the normality of the distribution of data. Quantitative parametric data were presented as mean and standard deviation (SD) and were analyzed by unpaired student t-test. Quantitative non-parametric data were presented as the median and interquartile range (IQR) and were analyzed using the Mann-Whitney test and the Friedman test for abnormally distributed quantitative variables, to compare between more than two periods, and post-hoc test (Dunn's) for pairwise comparisons. Qualitative variables were presented as frequency and percentage (%) and analyzed using the Chi-square test or Fisher's exact test when appropriate. A two-tailed P-value < 0.05 was considered statistically significant.

## Results

Table 1 shows that there were no significant differences between both groups regarding age, gender, occupation (housewife, manual worker, and others), medical history (free, hypertension [HTN], cardiac, and others), side affected (right, left, and bilateral), dominant hand (right and left), associated clinical finding of the hand (none, trigger finger, CTS [carpal tunnel syndrome], and osteoarthritis wrist), and previous intervention (NSAIDS, steroid injection [other side], and brace). Moreover, in group I, 15 patients were affected on the dominant side. Similarly, in group II, 14 patients were affected on the dominant side (Table 1).

**Table 1:** Patient characteristics: medical history, side affected, and dominant hand of the studied patients (n = 40) Data are presented as mean ± SD, median (IQR), or frequency (%). IQR: Interquartile range; HTN: Hypertension; NSAIDs: Non-steroidal anti-inflammatory drugs.

		Group I (n = 20)			Group II (n = 20)			P-value
Age (years)	Mean ± SD	42.30 ± 12.03			44.50 ± 11.84			0.563
	Median (IQR)	40.50 (31.5–54.5)			42.50 (38.5–52.5)			
Gender	Male	4 (20.0%)			3 (15.0%)			1.000
	Female	16 (80.0%)			17 (85.0%)			
Occupation	Housewife	12 (60.0%)			14 (70.0%)			0.710
	Manual worker	5 (25.0%)			5 (25.0%)			
	Others	3 (15.0%)			1 (5.0%)			
Medical history	Free	14 (70.0%)			16 (80.0%)			0.867
	HTN	3 (15.0%)			3 (15.0%)			
	Cardiac	1 (5.0%)			0 (0.0%)			
	Others	2 (10.0%)			1 (5.0%)			
Side affected	Right	15 (75.0%)			13 (65.0%)			0.487
	Left	4 (20.0%)			7 (35.0%)			
	Bilateral	1 (5.0%)			0 (0.0%)			
Dominant hand	Right	18 (90.0%)			19 (95.0%)			1.000
	Left	2 (10.0%)			1 (5.0%)			
		Right	Left	Bilateral	Right	Left	Bilateral	-
Side affected, dominant hand	Right	14 (77.8%)	3 (16.7%)	1 (5.6%)	13 (68.4%)	6 (31.6%)	0 (0.0%)	-
	Left	1 (50.0%)	1 (50.0%)	0 (0.0%)	0 (0.0%)	1 (100.0%)	0 (0.0%)	-
P-value		0.448			0.350			0.302
Associated clinical findings of the hand	None	18 (90.0%)			15 (75.0%)			
	Trigger finger	2 (10.0%)			1 (5.0%)			
	CTS (carpal tunnel syndrome)	0 (0.0%)			3 (15.0%)			
	Osteoarthritis wrist	0 (0.0%)			1 (5.0%)			
Previous interventions	NSAIDs	20 (100.0%)			20 (100.0%)			-
	Steroid injection (other side)	1 (5.0%)			0 (0.0%)			1.000
	Brace	2 (10.0%)			1 (5.0%)			1.000

There were statistically significant differences regarding VAS, QuickDash-9 score, and degree of disability. However, complications were statistically insignificant between both groups (P < 0.05). Nonetheless, two cases injected with CS had a post-injection flare. CS was better than PRP in two weeks, but PRP was superior to CS in six months with regard to QuickDASH-9 (P < 0.05). This difference was statistically significant (Table 2).

**Table 2:** Comparison between the two studied groups according to the VAS, QuickDash-9 scores, degree of disability, and complications Data are presented as mean ± SD, median (IQR), or frequency (%). VAS: Visual analog scale; IQR: Interquartile range. * Significant as P-value.

				Group I (n = 20)	Group II (n = 20)	P-value
VAS						
Before injection			Mean ± SD	7.40 ± 1.23	7.35 ± 1.23	0.841
			Median (IQR)	7.0 (6.5–8.5)	7.0 (6.0–8.5)	
After injection	2 weeks		Mean ± SD	6.0 ± 1.72	3.0 ± 2.0	<0.001*
			Median (IQR)	6.0 (4.50–7.5)	3.0 (2.0–3.50)	
	6 months		Mean ± SD	1.30 ± 1.63	5.55 ± 1.57	<0.001*
			Median (IQR)	1.0 (0.0–2.0)	5.0 (4.5–7.0)	
Improvement (before vs 2 weeks)				1.40 ± 0.99	4.35 ± 1.79	<0.001*
Improvement (before vs 6 months)				6.10 ± 1.77	1.80 ± 1.58	<0.001*
% of improvement (before vs 2 weeks)				19.82 ± 13.87	60.30 ± 25.81	<0.001*
% of improvement (before vs 6 months)				82.89 ± 20.25	23.91 ± 20.21	<0.001*
QuickDash-9 score						
Before injection			Mean ± SD	48.95 ± 15.37	52.07 ± 17.05	0.547
			Median (IQR)	54.20 (35.25–55.9)	55.20 (36.35–65.9)	
After injection	2 weeks		Mean ± SD	45.37 ± 15.30	20.77 ± 15.00	<0.001*
			Median (IQR)	49.10 (31.5–55.3)	14.15 (10.65–23.3)	
	6 months		Mean ± SD	15.74 ± 14.83	36.23 ± 19.95	<0.001*
			Median (IQR)	11.25 (9.1–16.0)	30.35 (20.3–51.5)	
Improvement (before vs 2 weeks)				3.59 ± 4.01	31.3 ± 16.7	<0.001*
Improvement (before vs 6 months)				33.22 ± 16.98	15.84 ± 13.31	0.001*
% of improvement (before vs 2 weeks)				7.64 ± 9.72	60.40 ± 22.27	<0.001*
% of improvement (before vs 6 months)				67.71 ± 22.04	32.96 ± 24.04	<0.001*
Degree of disability						
Before injection		Mild disability		3 (15.0%)	2 (10.0%)	0.530
		Moderate disability		13 (65.0%)	10 (50.0%)	
		Severe disability		4 (20.0%)	8 (40.0%)	
2 weeks		Mild disability		5 (25.0%)	16 (80.0%)	0.02*
		Moderate disability		12 (60.0%)	4 (20.0%)	
		Severe disability		3 (15.0%)	0 (0.0%)	
6 months		Mild disability		18 (90.0%)	6 (30.0%)	<0.01*
		Moderate disability		1 (5.0%)	12 (60.0%)	
		Severe disability		1 (5.0%)	2 (10.0%)	
Complications						
Uncomplicated				20 (100.0%)	18 (90.0%)	0.487
Complicated				0 (0.0%)	2 (10.0%)	

Before injection, both scores were similar. In group I, the VAS and QuickDASH-9 scores after two weeks decreased (P = 0.011, 0.001, respectively). However, after six months, the VAS and QuickDASH-9 scores significantly decreased (P = 0.001). Hence improvement was noticed in both periods but was more after six months. On the contrary, in group II, the mean VAS and QuickDASH-9 scores significantly decreased after two weeks. After six months, the VAS and QuickDASH-9 scores increased. Although the pain and satisfaction scores were better than the baseline score, their effect began to fade in the intermediate term (Table 3).

**Table 3:** Comparison between the three studied periods according to the VAS and QuickDASH-9 scores in each group Data are presented as mean ± SD, median (IQR), or frequency (%). VAS: Visual analog scale; IQR: Interquartile range. * Significant as P-value.

		Before injection	After injection		P	P1	P2	P3
			2 weeks	6 months				
VAS								
Group I (n = 20)	Mean ± SD	7.40 ± 1.23	6.0 ± 1.72	1.30 ± 1.63	<0.001*	0.011*	<0.001*	0.001*
	Median (IQR)	7.0 (6.5–8.5)	6.0 (4.50–7.5)	1.0 (0.0–2.0)				
Group II (n = 20)	Mean ± SD	7.35 ± 1.23	3.0 ± 2.0	5.55 ± 1.57	<0.001*	0.011*	<0.001*	0.003*
	Median (IQR)	7.0 (6.0–8.5)	3.0 (2.0–3.50)	5.0 (4.5–7.0)				
QuickDASH-9 score								
Group I (n = 20)	Mean ± SD	48.95 ± 15.37	45.37 ± 15.30	15.74 ± 14.83	<0.001*	0.018*	<0.001*	<0.001*
	Median (IQR)	54.20 (35.25–55.9)	49.10 (31.5–55.3)	11.25 (9.1–16.0)				
Group II (n = 20)	Mean ± SD	52.07 ± 17.05	20.77 ± 15.00	36.23 ± 19.95	<0.001*	<0.001*	0.009*	0.004*
	Median (IQR)	55.2 (36.35–65.9)	14.15 (10.65–23.3)	30.35 (20.3–51.5)				

There was no significant relation between age and final outcome six months post-injection in both groups (Table 4).

**Table 4:** Relation between age with improvement in the VAS and QuickDASH-9 scores after 6 months VAS: Visual analog scale.

	Improvement (before vs 6 months)			
	VAS		QuickDash score	
*Age (years)*	*Rs*	*P*	*Rs*	*P*
Group I (n = 20)	0.256	0.275	0.078	0.745
Group II (n = 20)	-0.130	0.586	0.001	0.997

There was no statistically significant relationship between sex, occupation, and side affected and final outcome in both groups (Table 5).

**Table 5:** Relation between sex, occupation, and side affected with improvement in the VAS and QuickDASH-9 scores after 6 months Data are presented as mean ± SD and median. VAS: Visual analog scale. * Significant as P-value.

Improvement (before vs 6 months)	Sex				P-value
	Male		Female		
Group I (n = 20)					
VAS	(n = 4)		(n = 16)		0.682
Mean ± SD	6.50 ± 1.29		6.0 ± 1.90		
Median (Min.–Max.)	6.50 (5.0–8.0)		6.0 (1.0–9.0)		
QuickDash score	(n = 4)		(n = 16)		0.335
Mean ± SD	26.85 ± 13.26		34.81 ± 17.80		
Median (Min.–Max.)	24.55 (15.80–42.50)		38.55 (1.55–66.05)		
Group II (n = 20)					
VAS	(n = 3)		(n = 17)		0.616
Mean ± SD	2.33 ± 2.08		1.71 ± 1.53		
Median (Min.–Max.)	3.0 (0.0–4.0)		1.0 (0.0–5.0)		
QuickDash score	(n = 3)		(n = 17)		0.179
Mean ± SD	27.33 ± 10.44		13.81 ± 12.95		
Median (Min.–Max.)	31.80 (15.40–34.80)		13.60 (0.0–36.90)		
Occupation					
	*Manual worker*	*Housewife*		*Others*	
*Group I (n = 20)*					
VAS	(n = 5)	(n = 12)		(n = 3)	0.462
Mean ± SD	6.40 ± 1.34	6.42 ± 1.51		4.33 ± 2.89	
Median (Min.–Max.)	7.0 (5.0–8.0)	6.50 (4.0–9.0)		6.0 (1.0–6.0)	
QuickDash score	(n = 5)	(n = 12)		(n = 3)	0.783
Mean ± SD	34.58 ± 11.52	33.92 ± 17.30		28.10 ± 27.97	
Median (Min.–Max.)	40.60 (15.80–42.80)	34.85 (1.55–66.05)		15.90 (8.30–60.10)	
*Group II (n = 20)*					
VAS	(n = 5)	(n = 14)		(n = 1#)	0.343
Mean ± SD	2.60 ± 2.07	1.57 ± 1.40		1.0	
Median (Min.–Max.)	3.0 (0.0–5.0)	1.50 (0.0–4.0)			
QuickDash score	(n = 5)	(n = 14)		(n = 1#)	0.391
Mean ± SD	23.12 ± 15.71	14.03 ± 12.23		4.70	
Median (Min.–Max.)	31.80 (0.0–35.40)	15.0 (0.0–36.90)			
Side affected					
	*Right*	*Left*		*Bilateral*	
*Group I (n = 20)*					
VAS	(n = 15)	(n = 4)		(n = 1#)	0.665
Mean ± SD	5.93 ± 1.94	6.50 ± 1.29		7.0	
Median (Min.–Max.)	6.0 (1.0–9.0)	6.50 (5.0–8.0)			
QuickDash score	(n = 15)	(n = 4)		(n = 1#)	0.530
Mean ± SD	30.84 ± 17.35	40.26 ± 17.60			
Median (Min.–Max.)	33.20 (1.55–60.10)	33.85 (27.30–66.05)		40.60	
*Group II (n = 20)*					
VAS	(n = 13)	(n = 7)		(n = 0)	0.643
Mean ± SD	1.85 ± 1.34	1.71 ± 2.06		–	
Median (Min.–Max.)	2.0 (0.0–4.0)	1.0 (0.0–5.0)		–	
QuickDash score	(n = 13)	(n = 7)		(n = 0)	0.588
Mean ± SD	14.75 ± 13.84	17.84 ± 13.07		–	
Median (Min.–Max.)	13.60 (0.0–36.90)	15.40 (0.0–35.40)		–	

## Discussion

In terms of pain relief, both groups had a similar pre-injection pain score as well as a mean of 7.40 ± 1.23 in group I and 7.35 ± 1.23 in group II. Both groups experienced significant improvement in pain after the injection. However, the improvement was more significant in the CS group during the first two weeks after the injection with a mean decrease (1.40 ± 0.99) in group I compared to a mean decrease (4.35 ± 1.79) in group II, which is a statistically significant difference according to chi-square. The PRP group showed a more significant improvement in pain relief after six months (↓6.10 ± 1.77) in group I and (↓1.80 ± 1.58) in group II. This difference in the improvement time frame may suggest that CS provided faster pain relief, while PRP injections provided longer-lasting pain relief.

We also compared the patient’s satisfaction between the two groups using the QuickDASH-9 score. The CS group showed more significant improvement in functional outcomes after two weeks compared to the PRP group, with a difference in the mean QuichDash-9 score from baseline of 3.59 ± 4.01 (7.64% ± 9.72) improvement in group I and 31.3 ± 16.7 (60.40% ± 22.27) in group II.

Nevertheless, group I showed a 33.22 ± 16.98 (↓67.71% ± 22.04) decrease in mean QuickDash-9 score versus ↓15.84 ± 13.31 (↓32.96% ± 24.04) improvement in group II. This denoted a significant improvement in functional outcomes after six months in the PRP group. This finding was consistent with the pain relief results and suggested that CS provided a more rapid improvement in function, while PRP injections provided long-lasting functional improvement.

Regarding complications, we found that both treatments were relatively safe and did not result in any major complications. However, we found that patients injected with CS were associated with two cases of post-injection flare. In contrast, PRP injections were not associated with any significant side effects. Factors that may affect the results were also assessed. However, there was no statistically significant correlation between the patient’s age, sex, occupation, or the side affected and the results of either CS or PRP injection with regard to VAS and QuickDash-9 scores.

We recruited 40 patients with a mean age of 43.40 ± 11.83 years (above 40 years). There was no significant age difference between the two groups (P = 0.563). Perhaps there was a better response to PRP in both groups among those aged 19-25 years, 26-35 years, 36-45 years, and >46 years. The patients were divided into two groups: group I received a single PRP injection, and group II received a single CS injection. Patients who were injected were instructed to wear a wrist brace. Most patients in both groups were females: 80% in group I and 85% in group II. The majority of patients were housewives, with 60% and 70% in group I and group II, respectively. There was no significant relationship between the dominant hand and the affected side.

Our findings are consistent with several studies, including a prospective study conducted by Gulati and Ramesh, which concluded that PRP injections were superior to CS injections in managing pain, improving finger motion, and reducing disability [14]. According to Ramesh et al., autologous PRP injection promoted histopromotion, which minimized the need for surgical intervention and reduced patient morbidity [15]. In 2020, El Sheikh et al. indicated that PRP outperformed CS in terms of pain alleviation and ultrasound findings in the intermediate term [16].

In a prospective study by Mardani-Kivi et al., 33 patients were injected with CS and found that mean VAS improved from 8.6 ± 1 to 1.3 ± 1.3 after three weeks, denoting the efficacy of CS injection in the short term [17]. Another study on the efficacy of PRP injections in musculoskeletal disorders by Ramesh et al. examined 141 patients and found a decrease in the mean VAS score from 9.42 to 3.42 after six months following a single PRP injection, implying how effective PRP was in the intermediate term [18].

Another cohort study by Debasish et al. to compare the efficacy of conservative, CS, and PRP injection in DQT revealed a good clinical outcome of PRP injection in the treatment of DQT. The study included 217 cases divided into three groups. They were only able to decrease the VAS scores of patients from 8.98 ± 0.57 to 4.91 ± 1.01 and 3.96 ± 1.94 at the one- and six-month follow-up periods. After a six-month follow-up, 30.43% of the CS group had a recurrence of symptoms compared to only 4.47% recurrence in the PRP group [19]. Another study by Giroti et al. found that the CS group had better pain relief and hand function tests at one-month and three-month follow-up, and the PRP group had better pain relief and hand function tests than the CS group after six months [20].

A prospective study conducted by Anandha et al. suggested that although steroid injection is a therapeutic option for DQT, open surgery appeared to be a more beneficial method with relatively low recurrence and complication rates [21]. On the other hand, some studies show that there was no significant difference between CS and PRP in DQT. For example, a recent study by Kumar et al. on 60 patients using DASH, VAS, and MAYO wrist scores showed no significant difference between functional outcomes in CS and PRP groups. However, statistically significant complications (P = 0.026) like subcutaneous fat atrophy, depigmentation, and temporary increase in pain were seen in eight patients in the CS group. They concluded that although CS and PRP are equally effective in managing DQT, PRP was considered the safer option [22].

In conclusion, DQT is a common condition that can significantly impact the quality of life of affected individuals. This study compared the efficacy of PRP and CS in managing DQT and found that both treatments were effective in providing short-term pain relief, immediately after two weeks. However, PRP was found to be more effective in the intermediate term, with significant improvement in pain and function up to six months post-injection.

We recommend that further research is needed to determine the optimal number of injections and their timing. In terms of future directions, several avenues could be explored. First, the mechanism of action of PRP in DQT is not well understood. Future studies should aim to elucidate the underlying mechanisms of PRP and explore its potential role in promoting tissue healing and regeneration. Second, more research is needed to evaluate the long-term safety and efficacy of PRP. Finally, studies should also investigate the cost-effectiveness of PRP compared to CS as this is an important consideration in clinical practice, with CS being effective for the short term and concurrent PRP covering the intermediate/long term.

Limitations

First, the sample size was relatively small. Although the results were statistically significant, a larger sample size would increase the statistical power and the generalizability of the findings. Second, the follow-up period was relatively short. A longer follow-up period would allow for a more thorough evaluation of the long-term efficacy of these treatments. Third, we only evaluated a single injection of PRP or CS, whereas multiple injections may be necessary for some patients. Lastly, we did not evaluate other outcome measures such as grip strength and range of motion, which could provide a more comprehensive assessment of the treatment efficacy. The procedure should be performed echo-guided, as it should be, to ensure that there is no difference in the injection site among all patients within the first compartment.

## Conclusions

CS is more effective than PRP in the short term (two weeks), and PRP is more effective in the intermediate term (six months). Both modalities are safe; however, PRP is relatively safer than CS.
